# A retrospective study exploring parents’ perceptions of their child’s assessment

**DOI:** 10.3389/fpsyg.2023.1271746

**Published:** 2024-02-12

**Authors:** Filippo Aschieri, Giulia Cera, Elisabetta Fiorelli, Sara Brasili

**Affiliations:** ^1^Department of Psychology, Università Cattolica del Sacro Cuore, Milan, Italy; ^2^Department of Child Neuropsychiatry, Fondazione Don Gnocchi, Milan, Italy

**Keywords:** clients’ perspective, family assessment, grounded theory, parents’ satisfaction, parental perceptions, parent–teacher relationship

## Abstract

The current study investigates parents’ perceptions of their child’s assessment, focusing on their responses to the Italian version of the Parents’ Experience of Assessment Scale (QUEVA-G). Twenty parents, who voluntarily agreed to be contacted after completing the questionnaire, participated in qualitative interviews to gain deeper insights into their assessment experiences. A thematic analysis was conducted on the interview transcriptions, highlighting three primary domains of parental experience: (1) parental perceptions of the assessment process; (2) effects of the assessment; and (3) parental perceptions of their relationship with their children’s teachers. The findings indicate that the QUEVA-G accurately captures most areas of interest as well as reveals unexplored aspects.

## Introduction

There is consensus among researchers in recognizing therapeutic alliance as a crucial element concurring to the success of treatments with patients of all ages (Elvins and Green, 2008). In adult psychotherapy, treatments are often conducted individually, whereas those of children and adolescents almost always involve other family members. Therefore, it is essential to work toward building multiple alliances between the therapist and the child, the therapist and the parents, and the parents and the child (Shelef et al., 2005; Robbins et al., 2006).

Establishing a positive and collaborative relationship with parents is essential for several reasons. Parents play a pivotal role in fostering an alliance between the child and the therapist (Kazdin et al., 2006; Campbell and Simmonds, 2011). In addition, they are involved in the definition of children’s motivation to accept assistance and stick to the treatment plan (Fields et al., 2004). Moreover, the literature highlights that the level of parental involvement in children’s therapy is associated with treatment outcomes as the active engagement of only one or both parents in their child’s therapy sessions is necessary for its success (Fields et al., 2004; Karver et al., 2018). Indeed, parental commitment, both within and outside of therapy sessions, facilitates therapeutic change in the child/adolescent (Kazdin et al., 2006; Marker et al., 2013). Kazdin et al. (2006) also highlighted that limited parental involvement reduces the likelihood of beneficial changes for the child.

Brodard et al. (2019) explored the connections between alliance, parental expectations, and the relationship with the psychologist in the context of the assessment of a child. They found general feedback from parents about the helpfulness and clarity of the assessment process to be positive and a generally higher involvement and motivation for change for mothers in the assessment process compared with fathers. Initially, parents wished to increase the understanding of their children and to learn how to improve their children’s behaviors at school. The alliance between the assessor, parents, and children increased during the assessment. The initial alliance between assessors and parents predicted the evaluation of the utility of the assessment at its conclusion, mediated by the final level of alliance between parents and assessors. Of interest, initial lower levels of perceived alliance between children and assessors from parents predicted higher levels of parental motivation for change and perception of the utility of the assessment at its end. These results highlight that alliance, expectations, and the quality of the experience of parents play a fundamental role in the assessment of children and their families.

This study presents the qualitative segment of a research project aiming at uncovering how parents perceive the psychological assessment process of their child and the factors that contribute to its evaluation through a mixed-method approach that integrates quantitative and qualitative data. Specifically, this article presents the follow-up of quantitative research that provided the psychometric proprieties of the Parent Experience of Assessment Scale (PEAS; Austin, 2011; Austin et al., 2016), in Italy (QUEVA-G; Aschieri et al., 2024). The QUEVA-G, which maps five important dimensions of psychological assessment in children and adolescents with their families according to the Therapeutic Assessment model (Tharinger et al., 2022), includes the following factors: Parent-Assessor Relationship and Collaboration (PARC), New Understanding of the Child (NUC), Child-Assessor Relationship (CAR), Systemic Awareness (SA), and Negative Feelings (NF). Parent-Assessor Relationship and Collaboration (7 items) refers to the extent to which parents, during the assessment process of their child, were able to perceive themselves as actively engaged and genuinely assisted by the assessor [e.g., “*I was informed about each step of the assessment*”]. New Understanding of the Child (5 items) assesses the potential for parents to develop more accurate narratives regarding their child’s issues and acquire more effective educational skills through the assessment process [e.g., “*I have lots of new ideas about how to parent my child*”]. The quality of the relationship between the child and the assessor, expressed in terms of empathy, support, and understanding, is investigated through the Child-Assessor Relationship factor (4 items) [e.g., “*My child felt comfortable with the assessor*”]. Systemic Awareness (4 items) focuses on the possibility that parents, through the assessment process, may arrive at a more systemic view of their child’s issues, thus perceiving that the entire family needs to make small changes to assist him or her [e.g., “*The assessment revealed how family members play a role in my child’s problems*”]. The extent to which parents have felt ashamed, blamed, or judged during the assessment is explored by the Negative Feelings subscale (4 items) [e.g., “*The assessment made me feel like a bad parent*”].

Typically, qualitative surveys are used to validate or develop appropriate quantitative instruments. However, in this project, a reverse approach was adopted. Initially, a quantitative investigation was conducted using the Italian version of the PEAS (QUEVA-G), followed by a qualitative exploration of some participants’ experiences. This approach bears some resemblance to that of assessors using Therapeutic Assessment (TA; Finn, 2007; Durosini and Aschieri, 2021; Aschieri et al., 2023), a semi-structured and brief therapeutic intervention grounded in psychological assessment, where qualitative aspects follow quantitative measures. After administering standardized tests, clinicians engage in an extended inquiry involving a semi-structured collaborative discussion with clients about their testing experiences. This unique approach enabled the identification of the unmet needs of parents and informed necessary changes to provide more satisfactory services that address the needs of all involved individuals.

### Aims

This study aimed to investigate the thoughts, feelings, and experiences that underlie the responses provided by participants to the items of QUEVA-G, hence providing qualitative information about parents’ experience of the assessment process and outcomes. The rationale of the study is to explore, starting from QUEVA-G scores, and without any fixed *a priori* hypothesis, the experiences of parents whose children participated in an assessment. The primary objective was to gain a comprehensive understanding of how parents perceive the psychological assessment of their child, specifically by exploring (1) the factors contributing to positive or negative assessment experiences, (2) which of these factors are addressed by QUEVA-G and which ones remain unexplored, and (3) the unmet parental needs concerning children’s and adolescents’ mental health services and practices.

## Method

### Participants

#### Recruitment

Participants were recruited as part of a research project using the Italian version (Aschieri et al., 2024) of the Parent Experience of Assessment Scale (PEAS; Austin, 2011). The sample consisted of parents whose children had undergone an assessment in the previous year—to ensure that the memory of the evaluation was still vivid—and had reported their experience using QUEVA-G. These parents also expressed their willingness to be contacted for a follow-up interview regarding their experience. No exclusion criteria were applied concerning the children’s diagnosis, their level of functioning, or the typology of assessment completed. The researchers contacted the parents who volunteered to participate, provided them with a detailed explanation of the study’s procedures, and obtained informed consent. All participants were Italian-speaking adults.

Among the initial pool of the previous study’s participants (Aschieri et al., 2024; *N* = 185), 53 parents (29.94%) indicated their availability to be contacted for this study at the end of the QUEVA-G administration. Through convenience sampling, we contacted potential participants to schedule the interview. In the process of data collection, five parents withdrew their availability to be interviewed. Altogether, 20 parents were interviewed. The sample size was motivated by the saturation of thematic categories. Generally, in qualitative research with homogeneous participants (i.e., parents whose children undergo psychological assessment) and a relatively narrow focus (i.e., the parents’ experiences of the assessment), the literature indicates an array of interviews ranging from 9 to 17 for data saturation (Hennink and Kaiser, 2022). Following Young and Casey (2019), saturation was defined using a “code frequency count” approach: Transcripts were read sequentially while counting the number of new codes that emerged from each interview until no more codes were identified. In our study, we reached a consensus that thematic saturation was achieved after interviewing 20 participants.

#### Sample characteristics

Twenty participants were interviewed, all of whom were biological mothers, with the exception of one grandmother. The majority of children and adolescents undergoing assessment were boys (*n* = 13; 65%). The age of the assessed children and adolescents ranged from 4 to 15 years, with a mean age of 8.9 years (SD = 3.15 years). In most cases (*n* = 14; 70%), the assessment focused on cognitive or neurodevelopmental disorders, particularly specific learning disabilities (SLD) and attention-deficit/hyperactivity disorder (ADHD). Additionally, 5% of the sample sought assessment for emotional or behavioral problems in their child (*n* = 1). In 15% of cases, the assessments addressed mixed concerns, involving both cognitive and emotional-behavioral aspects (*n* = 3). Finally, in 10% of cases, the type of assessment could not be clearly identified (*n* = 2).

### Instruments: the extended inquiry (EI)

Participants engaged in an extended inquiry (EI) during which they were asked about the responses they provided on the QUEVA-G The EI is a semi-structured collaborative discussion between the assessor and the client immediately after the test is administered. Its purpose is to gather information that may not be captured in the norm-based results by delving into the personal meaning behind the client’s responses. In an EI, assessors begin with general questions such as “*What was it like for you to complete this questionnaire?*” or “*Did you notice anything that caught your interest in any of the items you responded to?*” Following these questions, assessors shifted their focus to more specific topics, such as “*I observed that you did not answer all the items related to…*” or “*I noticed that your eyes became teary when you mentioned missing your mother deeply*.” This process facilitates a deeper understanding of how test responses and results align with the broader context of the clients’ lives. In the clinical setting, the EI encourages clients to establish their own connections, thereby enhancing their sense of self-efficacy and self-esteem (Fantini et al., 2022). Following up on clients’ observations and experiences about the testing often highlights relevant associations and unexpected themes that respondents find significant and related to their goals for the assessment.

During the interviews conducted in our study, two main themes were explored. First, we delved into participants’ general impressions and subjective evaluations of the questionnaire and how it related to their experience of the assessment. Following, we present an excerpt of this process from an interview (participant #6):

*Interviewer (I.): Thank you for agreeing to be interviewed. The first topic I’d like to discuss with you is, what was it like to reflect on the assessment while completing the QUEVA-G?*

Second, the interviews focused on relevant items, such as those scored at the extremes of the response scale (e.g., “*The assessment made me feel ashamed*,” 5—very much) or when respondents scored in opposite directions on similar items (e.g., “*Now I know what to expect from my child*,” 1—Not at all, and “*I understand my child so much better now*,” 5—very much). Following, we present an excerpt from another interview (participant #13):

*(I.): An aspect I noticed is that you indicated not feeling judged and not feeling ashamed during the assessment. The only item you scored high was “The assessment made me feel like a bad parent,” and that intrigued me because all the other scores are very low. So, I wanted to ask how is that?*

### Procedure

The study obtained institutional review board approval (Practice number: 42–23). Data collection took place between November 2022 and April 2023. The average duration of each interview was 43 min, with the maximum and minimum durations being approximately 75 and 19 min, respectively. Participants were given the option to choose between remote interviews via WhatsApp or Teams or in-person interviews. All interviews were audio recorded and subsequently transcribed.

### Data analysis

A thematic analysis (Braun and Clarke, 2006) was conducted on the interviews, focusing on identifying emerging recurring patterns of meanings represented as codes or subthemes. These codes were then organized into broader conceptual categories known as themes, and their interconnections were explored to construct an explanatory model.

The coding process was inspired by Braun and Clarke’s (2006) guidelines. While interviewing participants, researchers familiarized themselves with the data by thoroughly reading and re-reading the transcripts of the interviews to gain familiarity with the data. In this phase, interviewers started generating initial codes by associating them with specific segments of text. Individually and in group meetings, the authors developed a tentative grouping of these codes into potential themes, considering their conceptual coherence and continuity. The revision and refinement of the themes into a thematic map reflecting the collective data occurred once the saturation of codes was reached.

Eventually, the thematic map was defined through further revisions and enhancements.

The reliability of findings was ensured by a detailed report of transcripts pertaining to all codes (examples of all codes are presented in Appendix A). As in Aschieri et al. (2021), the trustworthiness of the results was supported by the analysis of notes written during and after debriefings among co-authors. Emotional reactions of interviewers facing the parents’ accounts of their children’s assessments were processed with the first author through debriefings. Throughout the research process, there was an ongoing reflexive dialogue between the researchers that constantly reflected on their own positioning, biases, and assumptions in relation to the phenomenon under investigation.

## Results

**Figure 1:**
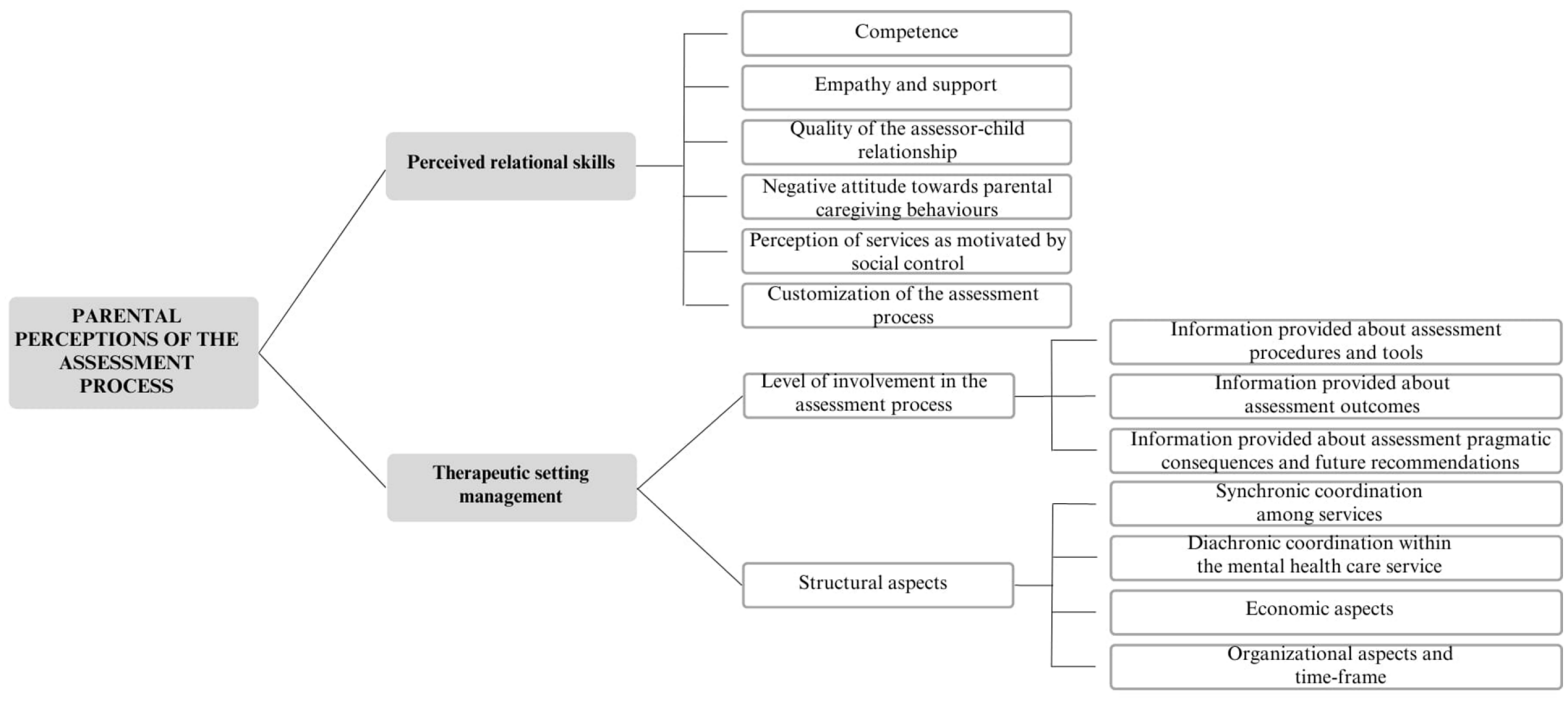
Parental perceptions of the assessment process.

**Figure 2:**
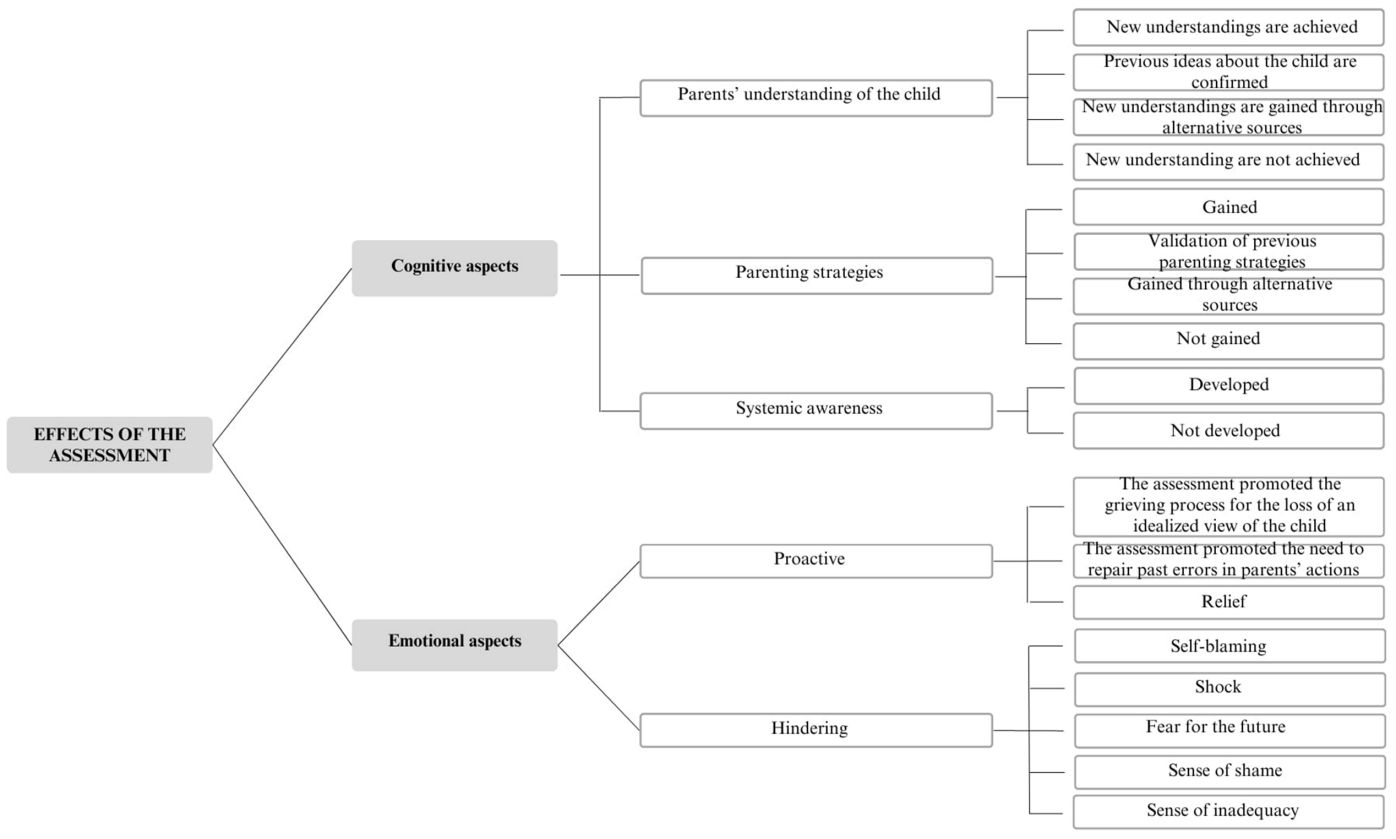
Effects of the assessment.

**Figure 3:**
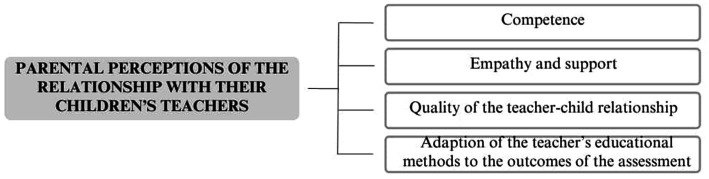
Parental perceptions of the relationship with their children’s teachers.

The final coding scheme comprised four levels of analysis: main themes, themes, secondary themes, and subthemes. The three main themes that emerged from the coding process were (1) parental perceptions of the assessment process (Figure 1), (2) effects of the assessment (Figure 2), and (3) parental perceptions of the relationship with their children’s teachers (Figure 3).

### Parental perceptions of the assessment process

The first main theme encompasses parents’ perceptions developed throughout the entire assessment process, encompassing their views of both the assessor and the mental healthcare agency. Particularly, parents’ perceptions of the assessor are influenced by various relational skills, such as competence and empathy. Moreover, parental perceptions of the assessor and the service are intertwined with the management of the therapeutic setting. This aspect is closely related to their level of involvement during the assessment and various structural aspects, including organizational and economic factors.

### Perceived relational skills

- Competence: Some participants expressed that their trust in the assessor was influenced by the assessor’s professionalism. For instance, P13 stated, «*During the speech therapy session, they mentioned that my child seemed to have dysgraphia issues because he could not draw well. They emphasized that, at his age, children should be able to draw certain things, and his geometries were not typical of a five-year-old. However, I asked them, “Did you inquire about what he was trying to draw?” and they responded with, “No, no”*».
- Empathy and support: Parents’ trust in the assessor was influenced by the assessor’s ability to empathize with them and provide support. P3 shared the following experience: «*Whenever I had any doubts or came across new information and asked for explanations, she was always very helpful. She explained what options would be beneficial for us and what might not work. She encouraged us to try different approaches because what works for one person may not work for another. I found her to be consistently positive and open to discussions. Initially, I had many questions, but she was always kind and supportive, even when helping us explore different methods*».
- Quality of the assessor–child relationship: Some participants indicated that their trust in the assessor was influenced by the assessor’s relationship with their child. For instance, P14 shared, «*My daughter and I are very pleased with the assessment. My niece always leaves with a happy mood, and when I ask her about her experience there, she always tells me how happy she is to go there*».
- Negative attitude toward parental caregiving behaviors: Some parents reported feeling criticized by the assessor and being accused of being the “cause” of their child’s problems. P1 shared, «*The doctor accused me of being too overprotective with my daughter; I felt like, “Oh my God, maybe I’m overreacting? Am I not seeing things clearly?” So, I started doubting myself. The same thing happened with the therapists at the private clinic I visited, where they said, “Stop medicalizing your daughter!”*».
- Perception of services as motivated by social control: Some participants expressed the belief that mental healthcare services might not have genuine intentions to assist them, leading to suspicions that these services could be driven by social control purposes. P19 stated, «*But also, for example, the mood stabilizer that the psychiatrist immediately prescribed… There are natural alternatives: natural mood-stabilizing solutions (…). So why do we rely on medications? Many parents are unaware of this, and what happens? They continue to administer drugs to their children (…) and the children keep gaining weight or remaining sedated. It’s like they want to sedate them, control them, and waste all the parents’ money*».
- Customization of the assessment process: Parental perceptions of their child’s evaluation were influenced by the assessor’s ability to create an individualized assessment path based on the specific needs of the family. P2 stated, «*The impression I had was that the assessor did her job in a very impersonal way, just reporting the evaluation, conducting the tests, and then “goodbye and thank you”*».

### Therapeutic setting management

Level of involvement in the assessment process:

- Information provided about assessment procedures and tools: Participants’ satisfaction with the assessment was influenced by the amount of information provided by the assessor about the assessment procedure, including the tools used and the steps followed. P13 mentioned, «*We left the child alone during the assessment, and in the end, we were unaware of how it went and what tests were conducted*».
- Information provided about assessment outcomes: Parents’ satisfaction with the assessment was linked to the amount of information provided by the assessor regarding the evaluation’s outcomes to gain a deeper understanding of their child’s difficulties. P1 expressed, «*At the time of receiving the functional diagnosis, I wanted to know as soon as possible what it meant to have a hyper-kinetic syndrome. I wondered, “Is this ADHD? Is it temporary? Will it pass? Was it caused by me or the school?”. I could not comprehend it fully, and it wasn’t explained in detail, so it caused me a lot of suffering*».
- Information provided about pragmatic consequences of the assessment and future recommendations: Parental satisfaction with the assessment was influenced by the level of information given by the assessor about post-assessment involvements and future interventions. P12 shared, «*They recommended that my daughter started a therapy because she is very emotional and has difficulty speaking in front of others. So, I was advised to start this process*».

Structural aspects:

- Synchronic coordination among services: Parental satisfaction was influenced by the ability of services (school and mental healthcare agencies) to interact with each other in delivering interventions. P11 explained, «*For example, the assessor wrote a report that we gave to the school (…), but there was no communication between her and the school. So, I ended up delivering the report to the teachers myself, and I do not even know if it would have been helpful for her to talk to the teachers, but she did not propose it, and they did not ask for it*».
- Diachronic coordination within the mental healthcare service: Parental satisfaction was influenced by the ability of mental healthcare agencies to provide continuous intervention to the family, enhancing their perception of being deeply supported. P2 stated, «*The assessment is a journey that should begin and continue, but instead, it ends with just a diagnosis sheet. As a parent, you feel lost, carrying this sheet and shouting, “Help! Help*!*”*».
- Economic aspects: Economic factors affected parents’ satisfaction with the received service in different ways, primarily depending on the financial situation of the family and the perceived utility of the assessment. P10 expressed, «*There is no adequate support from the National Health Service or the municipalities. For instance, municipalities are not interested because they have more serious cases to handle. They do not provide the Health Service bonus, claiming we are not entitled to it. But I cannot handle it alone… often, I need support, but I cannot afford it because I cannot pay for it…*».
- Organizational aspects and time frame: Parental satisfaction with the mental healthcare agency was influenced by the waiting time before receiving the assessment and the overall duration of the process. P13 shared, «*For our feedback, however, I waited for a year because the person who had initially tested my son – was she a PhD student? – had left, so the operators would have had to redo the entire evaluation. So, they simply looked at what she had written; there was no further exploration of my child’s aspects, and I could not ask why they told me so about my child because they could not answer*».

### Effects of the assessment

This second main theme deals with what the assessment process has provided to the family both cognitively and emotionally.

#### Cognitive aspects

Parents’ understanding of the child:

- New understandings are achieved: The assessment process facilitated a deeper and more nuanced parental understanding of the child. P4 stated, «*After the assessment, it’s like you are given a magnifying glass, and you can understand everything better. It’s like saying to someone who is blind, “Sorry, but you read, right? How can you not read?”. Well, I could not see that my child was blind and it did not make sense to ask her to try*».
- Previous ideas about the child are confirmed: The assessment process confirmed parents’ previous views about their child without adding new information. P5 mentioned, «*At least, maybe, the neuropsychiatrist told me that she is a sensitive child, just as the teachers have noticed before, telling me that she must feel supported… but I have already known this. They reported things that I had observed myself and that I correctly understood her*».
- New understandings are gained through alternative sources: Despite the assessment, some parents obtained a deeper understanding of their child through alternative sources rather than through the assessment process. They sought support from other parents facing similar difficulties, read articles about their children’s diagnosis online, or engaged with books and parents’ associations on the subject. P1 explained, «*So the information that I learned the most was through other parents, sharing experiences where you recognize yourself in the same problems, and I read articles about ADHD on the internet*».
- New understandings are not achieved: Some parents did not gain a deeper understanding of their child’s traits and difficulties through the assessment process. P11 expressed, «*The assessment was a mostly positive experience, but it did not provide all the answers or solve all the doubts… I still feel the need for a key to understand what lies behind the difficulties of my child*».

Parenting strategies:

- Gained: The assessment process enabled parents to acquire more effective parenting strategies to deal with their children. P8 stated, «*I understand now why my son does certain things… I did not understand his stereotypes before, and I used to correct them. Now, I know how to approach him when he engages in certain repetitive behaviors*».
- Validation of previous parenting strategies: The assessment process validated parents’ previous parenting strategies without suggesting new approaches. P6 mentioned, «*I asked the assessor for some suggestions, not about daily education but more related to everyday tasks. But, they mostly confirmed the things I was already doing*».
- Gained through alternative sources: Despite the assessment, some parents gained new strategies for dealing with their child through alternative sources rather than through the assessment process. They sought support from other parents, pursued information online, and engaged with relevant books and parents’ associations. P18 explained, «*I figured out how to handle it, but by reading and training. For example, everyone said, “Hold still. Sit still,” but if he does not manage it, we cannot keep telling him that. Instead, we can say, “Do not hurt your sister” (…). I trained myself, but nobody provided any guidance after the diagnosis*».
- Not gained: Some participants did not acquire new parenting strategies. P9 shared, «*The assessment did not help me at all. It did not help my son either…Its effectiveness was as transparent as air…It did not provide any helpful tools*».

Systemic awareness:

- Developed: Through the assessment process, parents gained awareness of their family’s influence on their children’s difficulties. P20 explained, «*My daughter’s dad lives in a family that’s a bit entangled. We are quite hypochondriacal and very anxious, and I believe that living with these dynamics sometimes particularly accentuates an anxious symptomatology in my daughter. She’s the only little niece in a family of older people, so she grew up in an environment where she is the center of the world, and this probably contributed to her immaturity from various points of view*».
- Not developed: Some parents were not able to recognize their impact on their children’s difficulties. P16 stated «*Irrespectively of the assessment, I do not think my son’s difficulties depend on family conflicts. I think he would have been the same anyway because even when he was a little boy, he was like that. So, I do not see a correlation*».

#### Emotional aspects

Proactive:

- The assessment promoted the grieving process for the loss of an idealized view of the child: During the assessment, parents developed the ability to embrace their children’s unique traits and put themselves in their shoes. P12 explained, «*I learned to try to put myself in my children’s shoes, which is sometimes difficult, and to accept them as they are. Before, I used to get angry if they got a bad grade or if I thought the mark was not what they were supposed to get. But now, I have learned to accept them as they are and understand that they have some difficulties, and some may have more or fewer difficulties, and to accept them as they are*».
- The assessment promoted the need to repair past errors in parents’ actions: Parents felt guilt for their past actions with their children, and this motivated them to make positive changes and create a more comfortable environment for their children. P2 shared, «*I allowed my husband to do many things that maybe I should not have allowed, and that marked my son’s childhood. Not anything dramatic, but phrases that I might have noticed the impact they could have on him. So… I could have done more, but maybe it’s a feeling that all parents have, or at least those who question themselves*».
- Relief: The assessment process resulted in a confirmation of the parents’ suspicion that something was going on with their children, leading to a deeper sense of wellbeing for the entire family. P19 said, «*In about 80% of the cases, the assessment comes when the parent now has awareness that something is wrong. Before this, most of the time, they treat you like you are overreacting, but when the evaluation comes, it gives you a sense of relief. It’s like saying, ‘Gosh, it’s not that I’m wrong, it’s not my daughter who’s wrong. We were not wrong when thinking about an assessment to figure out what was happening*».

Hindering:

- Self-blaming: Some parents criticized themselves, believing themselves to be the “cause” of their children’s difficulties. P18 shared, «*At first, when my son was diagnosed, I thought I might have given him this negative gene*».
- Shock: Some parents experienced traumatic feelings upon discovering their children’s diagnosis, such as desperation and hopelessness. P8 explained, «*When you get these things, it’s like a cold shower, and you think, “Why me? Why him? Why us?”*».
- Fear for the future: Some parents did not know what to expect for their child’s future, such as how their difficulties might evolve or who could help them. P13 expressed, «*The problem is the uncertainty about what happens next because nobody knows what to do with it. It’s not like having a problem with a solution, especially in the case of intellectual giftedness. So, what do you do? What do you do with the child’s relationships? There is no specific intervention option, such as speech therapy used for learning disabilities, for example*».
- Sense of shame: Some parents felt ashamed of themselves because of their children’s difficulties or for not having discovered their diagnosis earlier. P4 shared, «*Not recognizing the pathology in him and not being able to understand it made me feel ashamed, to be honest. I felt ashamed that I did not get it, that I yelled at him, gave him a smack, and said, “How can you not understand multiplication tables?”*».
- Sense of inadequacy: Some participants felt like “bad parents” because of their lack of knowledge about their children’s diagnosis and their incapacity to discover it earlier. P20 expressed, «*I think of myself as a bad parent because when I had the first hints, I should have acted immediately. Instead, it took me a year… before getting my daughter evaluated. I feel a little guilty because it took me a while to start*».

### Parental perceptions of the relationship with their children’s teachers

This main theme comprises four variables that influence how parents perceive their child’s teachers and their relationship.

- Competence: Some parents expressed that their interactions with teachers were influenced by the educators’ expertise and theoretical knowledge in recognizing and addressing children’s difficulties. Participant 3 stated, «*Unfortunately, they are not really prepared, or perhaps not at all, to identify difficulties that go beyond the usual ‘he is lazy, he is listless, maybe he has difficulty in some school subject.’ In fact, there was something else: there was a learning disorder*».
- Empathy and support: Certain parents felt that the quality of their relationship with teachers depended on the educators’ ability to empathize with and support both the parents and their children. Participant 4 shared, «*I communicated with the teacher, saying, “My daughter believed she was assisting her!” and the teacher replied, “I cannot attend to twenty boys: the others can manage on their own.” That’s when I became upset. I’m glad that others may succeed, but she cannot do it alone*».
- Quality of the teacher–child relationship: The parent–teacher relationship was influenced by how parents perceived the teacher’s interactions with their child. P19 recalled, «*My daughter recalls her school experience as a period of being bullied by her teachers, not by her classmates. She was told things like “You’re not good, you are not committed, you are stupid” despite having a memory disorder and a very serious learning disability*».
- Adaptation of the teacher’s educational methods to the outcomes of the assessment: The parent–teacher relationship was affected by how teachers adjusted their educational methods based on the child’s specific needs identified during the assessment process. Participant 6 explained, «*In the second year, after I clarified things and informed them [the teachers] that I would soon have my son undergo an evaluation, there was a noticeable change. They seemed to become more aware of his difficulties. I kept them informed about all the steps we took during the process. Afterwards, they began approaching us differently: when I shared the diagnosis with them, everything changed*».

## Discussion

The thematic analysis has yielded a comprehensive set of results, revealing multiple factors that influence the quality of parents’ experiences during their child’s assessment. These findings align with a multidimensional view of the satisfaction construct, which is consistent with previous research on satisfaction (Donabedian, 1988; Rouse et al., 1994). The results of this study also align with the dimensions investigated by the QUEVA-G scale in accordance with the theoretical principles of Therapeutic Assessment. However, some new themes have emerged from this research that are not addressed by the scale.

### Parental perceptions of the assessment process

The results highlight the crucial role played by the relationship with the assessor in shaping parental experiences during their child’s assessment process. Participants expressed a more positive view of the assessment when they perceived the assessor as a reliable and competent figure who genuinely cared about helping them and addressing their concerns. Additionally, feeling understood, heard, and respected by the assessor contributed to a positive experience. This aspect aligns with the theoretical principles of Therapeutic Assessment: According to Finn’s (2007) perspective, establishing a positive and secure relationship between the assessor and parents promotes parental satisfaction and encourages their active participation throughout the evaluation process. Furthermore, parents emphasized the importance of the clinician building an excellent relationship not only with them but also with their children. On the other hand, negative experiences were reported when parents felt criticized for their caregiving behavior, perceived the clinician’s approach as impersonal, or believed that the clinician was more focused on exercising social control than genuinely helping them.

The management of the therapeutic setting by the assessor and the level of parental involvement during the evaluation process also strongly influenced their experience. Providing detailed information about the assessment process, its different phases, tools used, evaluation results, and future interventions was essential for parents. In contrast, inadequate provision of such information led to feelings of frustration, helplessness, and disorientation as parents were unable to fully comprehend their child’s special needs or make informed decisions regarding their child’s mental healthcare. These results further support the Therapeutic Assessment perspective as it encourages complete parental involvement and collaboration with the assessor throughout the evaluation process (Finn, 2007).

Although not investigated by the QUEVA-G scale, structural aspects of the evaluation process were found to be crucial in shaping parental experiences. Participants expressed frustration regarding the expensive cost of many private services, the lengthy waiting periods, and the high turnover of clinical staff. Moreover, effective coordination between different services, such as mental health services and schools, as well as between professionals involved in the evaluation and therapeutic phases, was crucial for successful outcomes. Collaborative efforts among different actors can lead to more synergistic and integrated interventions, promoting better wellbeing for the child. In contrast, a lack of proper coordination among services can result in misunderstandings, disconnections, and delays in the child’s care journey.

### Effects of the assessment

The main theme related to the effects of the assessment is well explored by the QUEVA-G scale although some subthemes are less considered. Assessment is particularly appreciated when it helps parents develop more accurate and empathetic narratives about their child, promote more functional family interactions, and foster a more systemic understanding of their child’s difficulties (Finn, 2007; Tharinger et al., 2009; Aschieri et al., 2013; Frackowiak et al., 2015). The items of the New Understanding of the Child and the Systemic Awareness subscales in QUEVA-G investigate these phenomena, referred to as “cognitive aspects.”

The other aspect of the main theme pertains to the emotional experience of parents during the evaluation process, encompassing both positive and negative emotions. The “Negative Feelings” subscale of QUEVA-G, as the name implies, only assesses the presence of negative emotions without delving into the plausible fear parents might have for their children’s future.

However, the QUEVA-G does not explore the positive feelings associated with the assessment process. In these interviews, the participants indicate that the evaluation frequently leads parents to experience a sense of reparative guilt, which can be instrumental in driving positive changes for the entire family. Additionally, the symbolic process of mourning connected to the child’s diagnosis—characterized by various stages, starting with denial and anger and progressing through bargaining and depression until acceptance—and the relief that it eventually brings represent crucial themes, even though they are not addressed by the scale, in assessments involving parents. Indeed, as Mazzoncini and Musatti (2012) pointed out, a developmental disorder is often experienced by parents as a form of mourning not only associated with the loss of their child’s skills and competencies but also involving a simultaneous loss of the ideal child and their own self-image as parents capable of raising a child without difficulties. Furthermore, this revelation is typically accompanied by a profound sense of guilt as parents frequently search for possible causes in their own behaviors. Therefore, it becomes the responsibility of the assessor to assist parents in correctly interpreting the assessment results and to support them in the process of understanding their child’s difficulties while also providing space for the anxieties and fears that inevitably emerge.

### Parental perceptions of the relationship with their children’s teachers

Unexplored by the QUEVA-G, the parental relationship with their child’s teachers appears to play a pivotal role in our participants’ experiences. The children of these participants have exhibited various developmental difficulties that can potentially impact their academic journey within the school system. Consequently, these parents are highly engaged in their interactions with teachers, finding greater satisfaction with the assessment process when teachers demonstrate unwavering commitment and genuine interest in assisting their child as well as providing personalized attention to their educational needs.

To facilitate the academic progress of these children and adolescents within the school system, Tharinger et al. (2011) proposed an intriguing application of Therapeutic Assessment techniques in the school environment. This approach emphasizes involving teachers as active participants in their students’ evaluations and supporting their curiosity, thereby fostering a sense of relevance in the child’s life. Consequently, teachers are more inclined to embrace the suggested recommendations and gain fresh perspectives in understanding the child’s difficulties. However, altering educational strategies in response to assessment findings can be challenging for teachers, who must balance their dedication to a single student with their broader responsibilities to the entire class (Mazzoncini and Musatti, 2012). To address these concerns and considerations, proposing focused intervention sessions and tailoring recommendations based on the individual teacher’s resources and the specific school context can be beneficial. This approach, as emphasized by Tharinger et al. (2011), has the potential to bring about concrete improvements in the lives of all parties involved.

### Implications for clinical practice, limitations, and future directions

To ensure parents’ satisfaction with their child’s psychological assessment, clinicians should consider the following guidelines: (1) consistently provide support to parents; (2) actively involve parents in the assessment process; (3) promote positive emotional and cognitive changes for both parents and children; (4) minimize waiting lists for assessments; (5) ensure that services are easily accessible and affordable; and (6) ensure continuity and coordination in service provision.

The significance parents place on teachers’ role in their child’s education has practical implications. Parents appreciate when teachers are trained to recognize and acknowledge their child’s disorders.

The teacher’s response to the communication of a child’s diagnosis by parents also holds importance. Parents find it important that assessors directly communicate assessment findings to the school and are engaged in translating assessment findings into educational strategies for the children.

The findings of this study should be considered while acknowledging its limitations. First, expanding the sample to include male participants would provide insights into fathers’ perceptions as well. The limitation of having a sample composed solely of female respondents is relevant even in the context of a qualitative study like this. While we searched for saturation of the thematic categories, conducting interviews with new participants until new themes emerged, the inclusion of fathers could have offered distinct perspectives and categories.

However, the absence of fathers in our sample can not only be considered a representative of the current cultural reality in many Western societies, where mothers often still bear the primary responsibility for childcare, but also aligns with findings from the literature. In Tiano et al.’s (2013) study, for instance, it was not possible to find any significant effect for the association between paternal involvement and acceptance of the proposed treatment (PCIT), primarily because the fathers in the sample spent significantly less time with their children than mothers. In this regard, in a recent review by Jukes et al. (2022), the authors attempted to identify potential gender differences in facilitators and barriers to parental engagement in their child’s treatment: While mothers reported obstacles relating to competing demands (e.g., housework and caregiving for sick relatives), fathers regarded seeking help as a sign of weakness and were less inclined to engage when they did not see themselves as primary caregivers or when their involvement conflicted with their ‘provider’ role.

## Conclusion

This study aimed to enhance our comprehension of the factors contributing to parental satisfaction with their child’s assessment by exploring with 20 qualitative interviews the experience of caregivers who volunteered to participate in the study. Despite the lack of generalizability of our results, participants’ voices have provided useful insights to understand which aspects of the assessment delivery process matter most to parents.

The first objective of the study was to investigate the factors contributing to positive or negative assessment experiences. According to the existing literature, the quality of the parent–assessor relationship emerges as the main factor in defining the parental experience of their child’s assessment, thereby exerting a significant influence on their care trajectory. However, other aspects need to be considered, including the opportunity for parents to gain a deeper understanding of their child’s problems, the feelings related to the evaluation process, satisfaction pertaining to structural components (such as economic factors, wait period, and coordination among services), and the role of the school in this context.

The second aim of this study was to identify which of these factors are addressed by QUEVA-G and which ones remain unexplored. Altogether, most of the identified codes are mapped by QUEVA-G (Aschieri et al., 2024), thus proving to be a more valuable and effective tool to investigate parental experiences than traditional measures such as CSQ. However, the structural aspects of the assessment process and the role of schools and teachers within the child’s journey of care are not considered by the QUEVA-G but have a significant impact on our participants’ narrations. Research about customers’ satisfaction has already investigated the structural aspects of service delivery. Our results suggest that the measurement of parental satisfaction with the assessment should also include the relationship between the parents and the school, and among these and the assessors. Parents’ relationship with their children’s teachers is strictly linked to the assessment process. Indeed, while recalling the evaluation experience, parents have almost always mentioned the school.

Finally, the third objective of this study was to identify the unmet parental needs regarding children’s and adolescents’ mental health services and practices. Each parent’s needs are different and unique, but our results demonstrate that some necessities are demanded by multiple participants. For instance, several parents stressed the importance of effective communication regarding their child’s diagnosis by the assessor to better understand his/her behavior and respond appropriately. In addition, participants mentioned the desire that clinicians might be able to serve as a bridge between them and the school system, thus helping teachers to better understand and respond to the child’s special needs.

The inclusion of a qualitative section in our research brought to light new perspectives, allowing us to gain a more comprehensive and nuanced understanding of our sample’s parental perceptions. Finally, this study represents an initial step to explore (also using a mixed-method approach, Creswell and Clark, 2017) the factors that may affect parents’ experience of their child’s assessment. In addition, it lays the groundwork for the development of new satisfaction measures that can consider those aspects that, even if important to parents, are not to date addressed by the existing tools (e.g., the relationship between services and schools).

## Data availability statement

The raw data supporting the conclusions of this article will be made available by the authors, without undue reservation.

## Ethics statement

The studies involving humans were approved by the Catholic University of the Sacred Heart. Number of institutional review board approval: 42–23. The studies were conducted in accordance with the local legislation and institutional requirements. The participants provided their written informed consent to participate in this study. Written informed consent was obtained from the individual(s) for the publication of any potentially identifiable images or data included in this article.

## Author contributions

FA: Methodology, Conceptualization, Funding acquisition, Resources, Supervision, Validation, Writing – review & editing. GC: Methodology, Data curation, Formal analysis, Investigation, Software, Writing – original draft. EF: Data curation, Supervision, Writing – review & editing. SB: Data curation, Formal analysis, Investigation, Methodology, Software, Writing – original draft.

## References

[ref022] Aschieri F. Brasili S. Cavallini A. Cera G. (2024). Psychometric Properties of the Italian Version of the Parent Experience of Assessment Scale. Front in Psychol. 14:1271713. doi: 10.3389/fpsyg.2023.1271713 PMC10868526 38362523

[ref1] Aschieri F. Barello S. Durosini I. (2021). “Invisible voices”: a critical incident study of family caregivers’ experience of nursing homes after their elder relative’s death. J. Nurs. Scholarsh. 53, 65–74. doi: 10.1111/jnu.12610, PMID: 33206459

[ref2] Aschieri F. Fantini F. Bertrando P. (2013). Therapeutic assessment with children in family therapy. Aust. N. Z. J. Fam. Ther. 33, 285–298. doi: 10.1017/aft.2012.37

[ref3] Aschieri F. van Emmerik A. A. P. Wibbelink C. J. M. Kamphuis J. H. (2023). A systematic research review of collaborative assessment methods. Psychotherapy 60, 355–369. doi: 10.1037/pst0000477 36972083

[ref4] Austin C. A. (2011). Investigating the mechanisms of therapeutic assessment with children: development of the Parent Experience of Assessment Scale (PEAS) (Doctoral dissertation). Retrieved from the University of Texas at Austin Texas Scholar Works. (Accession No. 2011-10-21T14:10:27Z).

[ref5] Austin C. A. Finn S. F. Keith T. Z. Tharinger D. J. Fernando A. D. (2016). The parent experience of assessment scale (PEAS): development and relation to parent satisfaction. Assessment 25, 929–941. doi: 10.1177/1073191116666950, PMID: 27630203

[ref6] Braun V. Clarke V. (2006). Using thematic analysis in psychology. Qual. Res. Psychol. 3, 77–101. doi: 10.1191/1478088706qp063oa

[ref7] Brodard F. Giroudeau I. Quartier V. Roman P. (2019). La perception parentale de la pratique du bilan psychologique de l’enfant et de l’adolescent. Prat. Psychol. 25, 383–397. doi: 10.1016/j.prps.2018.08.003

[ref8] Campbell A. Simmonds J. (2011). Therapist perspectives on the therapeutic alliance with children and adolescents. Couns. Psychol. Q. 24, 195–209. doi: 10.1080/09515070.2011.620734

[ref9] Creswell J. W. Clark V. L. P. (2017). Designing and conducting mixed methods research. Thousand Oaks, CA: Sage Publications, Inc.

[ref10] Donabedian A. (1988). The quality of care: how can it be assessed? J. Am. Med. Assoc. 260, 1743–1748. doi: 10.1001/jama.260.12.1743 3045356

[ref11] Durosini I. Aschieri F. (2021). Therapeutic assessment efficacy: a meta-analysis. Psychol. Assess. 33, 962–972. doi: 10.1037/pas0001038 34516194

[ref12] Elvins R. Green J. (2008). The conceptualization and measurement of therapeutic alliance: an empirical review. Clin. Psychol. Rev. 28, 1167–1187. doi: 10.1016/j.cpr.2008.04.002, PMID: 18538907

[ref13] Fantini F. Aschieri F. Finn S. E. (2022). Therapeutic assessment with adults. Using psychological testing to help clients change. New York: Routledge.

[ref14] Fields S. Handelsman J. Karver M. S. Bickman L. (2004). Parental and child factors that affect the therapeutic alliance. Paper presented at the 17th annual meeting of the Florida Mental Health Institute’s a System of Care for Children’s Mental Health: expanding the research base, Tampa, FL.

[ref15] Finn S. E. (2007). In our clients’ shoes: theory and techniques of therapeutic assessment. New York: Routledge.

[ref17] Frackowiak M. Fantini F. Aschieri F. (2015). L’évaluation thérapeutique: description de quatre modèles. Prat. Psychol. 21, 319–330. doi: 10.1016/j.prps.2015.09.006

[ref18] Hennink M. Kaiser B. N. (2022). Sample sizes for saturation in qualitative research: a systematic review of empirical tests. Soc. Sci. Med. 292:114523. doi: 10.1016/j.socscimed.2021.114523, PMID: 34785096

[ref19] Jukes L. M. Di Folco S. Kearney L. Sawrikar V. (2022). Barriers and facilitators to engaging mothers and fathers in family-based interventions: a qualitative systematic review. Child Psychiatry Hum. Dev. doi: 10.1007/s10578-022-01389-6, [Online ahead of print] PMC10796537 35763177

[ref20] Karver M. S. De Nadai A. S. Monahan M. Shirk S. R. (2018). Meta-analysis of the prospective relation between alliance and outcome in child and adolescent psychotherapy. Psychotherapy 55, 341–355. doi: 10.1037/pst0000176, PMID: 30335449

[ref21] Kazdin A. E. Whitley M. Marciano P. L. (2006). Child-therapist and parent therapist alliance and therapeutic change in a treatment referred for oppositional, aggressive, and antisocial behaviour. J. Child Psychol. Psychiatry 47, 436–445. doi: 10.1111/j.1469-7610.2005.01475.x, PMID: 16671927

[ref22] Marker C. D. Comer J. S. Abramova V. Kendall P. C. (2013). The reciprocal relationship between alliance and symptom improvement across the treatment of childhood anxiety. J. Clin. Child Adolesc. Psychol. 42, 22–33. doi: 10.1080/15374416.2012.723261, PMID: 23009693 PMC4224949

[ref23] Mazzoncini B. Musatti L. (2012). I disturbi dello sviluppo: bambini, genitori e insegnanti. (Milan: Raffaello Cortina).

[ref24] Robbins M. S. Liddle H. A. Turner C. W. Dakof G. A. Alexander J. F. Kogan S. M. (2006). Adolescent and parent therapeutic alliances as predictors of dropout in multidimensional family therapy. J. Fam. Psychol. 20, 108–116. doi: 10.1037/0893-3200.20.1.108, PMID: 16569095

[ref25] Rouse L. W. MacCabe N. Toprac M. G. (1994). Measuring satisfaction with community-based services for severely emotionally disturbed children: a comparison of questionnaires for children and parents. VII annual research conference for a “system of care” for children's mental health: expanding the research base, Tampa, FL.

[ref26] Shelef K. Diamond G. M. Diamond G. S. Liddle H. A. (2005). Adolescent and parent alliance and treatment outcome in multidimensional family therapy. J. Consult. Clin. Psychol. 73, 689–698. doi: 10.1037/0022-006X.73.4.689, PMID: 16173856

[ref27] Tharinger D. J. Finn S. E. Gentry L. Hamilton A. M. Fowler J. L. Matson M.. (2009). Therapeutic assessment with children: a pilot study of treatment acceptability and outcomes. J. Pers. Assess. 91, 238–244. doi: 10.1080/00223890902794275 19365764

[ref28] Tharinger D. J. Krumholz L. S. Austin C. A. Matson M. (2011). “The development and model of therapeutic assessment with children: application to school-based assessment” in The Oxford handbook of school psychology. eds. Bray M. A. Kehle T. J. (Oxford: Oxford University Press), 224–259.

[ref29] Tharinger D. J. Rudin D. I. Frackowiak M. Finn S. E. (2022). Therapeutic assessment with children: enhancing parental empathy through psychological assessment. New York: Routledge.

[ref30] Tiano J. D. Grate R. M. McNeil C. B. (2013). Comparison of mothers’ and fathers’ opinions of parent-child interaction therapy. Child Family Behav. Ther. 35, 110–131. doi: 10.1080/07317107.2013.789358

[ref31] Young D. S. Casey E. A. (2019). An examination of the sufficiency of small qualitative samples. Soc. Work. Res. 43, 53–58. doi: 10.1093/swr/svy026

