# Supplementary material for: A retrospective study exploring parents’ perceptions of their child’s assessment
Source: Front Psychol. 2024 Feb 12;14:1271746. doi: 10.3389/fpsyg.2023.1271746 (PMC10897758; doi:10.3389/fpsyg.2023.1271746)
Supplement: Supplementary file 1 [file Data_Sheet_1.docx]

**Appendix A** – **Report of Verbatim and Codes**

**Table 1 - Parental perceptions of the assessment process: Perceived relational skills**

| **Subthemes** | **Testimonials** |
| --- | --- |
| Competence | P15: *"The main thing trust in their expertise: they are highly knowledgeable, and I cannot emphasize enough how comfortable I feel there. It's a medical practice where, of course, reports and assessments on the child are prepared, but I have complete trust."* |
| Empathy and support | P3: *“Whenever I had any doubts or came across new information and asked for explanations, she was always very helpful. She explained what options would be beneficial for us and what might not work. She encouraged us to try different approaches because what works for one person may not work for another. I found her to be consistently positive and open to discussions. Initially, I had many questions, but she was always kind and supportive, even when helping us explore different methods”.* |
| Quality of the assessor-child relationship | P9: *"But he really didn't like the evaluation. In fact, at one of the last meetings, my son didn't speak... he was somewhat complaining, 'I don't want to go.'"* |
| Negative attitude towards parental caregiving behaviours | P1: “*The doctor accused me of being too overprotective with my daughter; I felt like, ‘Oh my God, maybe I'm overreacting? Am I not seeing things clearly?’ So, I started doubting myself. The same thing happened with the therapists at the private clinic I visited, where they said, ‘Stop medicalizing your daughter!’”.* |
| Perception of services as motivated by social control | P19: *“But also, for example, the mood stabilizer that the psychiatrist immediately prescribed... There are natural alternatives: natural mood-stabilizing solutions (...). So why do we rely on medications? Many parents are unaware of this, and what happens? They continue to administer drugs to their children (...) and the children keep gaining weight or remaining sedated. It's like they want to sedate them, control them, and waste all the parents' money”.* |
| Customization of the assessment process | P16:*“Because in my opinion, he was simply going through his routine, asking his routine questions."* |

**Table 2** **- Parental perceptions of the assessment process: Therapeutic setting management**

| **Secondary themes** | **Subthemes** | **Testimonials** |
| --- | --- | --- |
| Level of involvement in the assessment process | Information provided about assessment procedures and tools | P13: “*We left the child alone during the assessment, and in the end, we were unaware of how it went and what tests were conducted*”. |
|  | Information provided about assessment outcomes | P1: “*At the time of receiving the functional diagnosis, I wanted to know as soon as possible what it meant to have a hyper-kinetic syndrome. I wondered, “Is this ADHD? Is it temporary? Will it pass? Was it caused by me or the school?”. I couldn't comprehend it fully, and it wasn't explained in detail, so it caused me a lot of suffering*”. |
|  | Information provided about assessment pragmatic consequences and future recommendations | P12: “*They recommended that my daughter started a therapy because she is very emotional and has difficulty speaking in front of others. So, I was advised to start this process*”. |
| Structural aspects | Synchronic coordination among services | P10: *"The teachers have also been contacted by the speech therapist and the neuropsychiatrist... I must say that the specialists are very involved and proactive in this regard."* |
|  | Diachronic coordination within the mental health care service | P19: "*In my opinion, the major issue is the diagnosis and the agencies because, after all, even when your child is autistic, they simply say 'thank you and goodbye'."* |
|  | Economic aspects | P10: “*There is no adequate support from the National Health Service or the municipalities. For instance, municipalities are not interested because they have more serious cases to handle. They don't provide the Health Service bonus, claiming we're not entitled to it. But I cannot handle it alone... often, I need support, but I cannot afford it because I cannot pay for it…*”*.* |
|  | Organizational aspects | P13: “*For our feedback, however, I waited for a year because the person who had initially tested my son – was she a PhD student? – had left, so the operators would have had to redo the entire evaluation. So, they simply looked at what she had written; there was no further exploration of my child's aspects, and I could not ask why they told me so about my child because they could not answer*”. |

**Table 3 - Effects of the assessment: Cognitive aspects**

| **Secondary themes** | **Subthemes** | **Testimonials** |
| --- | --- | --- |
| Parents’ understanding of the child | New understandings are achieved | P4: “*After the assessment, it's like you're given a magnifying glass, and you can understand everything better. It's like saying to someone who is blind, “Sorry, but you read, right? How can you not read?”... Well, I couldn't see that my child was blind and it did not make sense to ask her to try*”. |
|  | Previous ideas about the child are confirmed | P5: “*At least, maybe, the neuropsychiatrist told me that she is a sensitive child, just as the teachers have noticed before, telling me that she must feel supported... but I have already known this. They reported things that I had observed myself and that I correctly understood her*”. |
|  | New understandings are gained through alternative sources | P1: *“So the information that I learned the most was through other parents, sharing experiences where you recognize yourself in the same problems, and I read articles about ADHD on the internet”.* |
|  | New understandings are not achieved | P11: “*The assessment was a mostly positive experience, but it didn't provide all the answers or solve all the doubts... I still feel the need for a key to understand what lies behind the difficulties of my child*”. |
| Parenting strategies | Gained | P8: “*I understand now why my son does certain things... I didn't understand his stereotypes before, and I used to correct them. Now I know how to approach him when he engages in certain repetitive behaviors*”. |
|  | Validation of previous parenting strategies | P6: *“I asked the assessor for some suggestions, not about daily education, but more related to everyday tasks. But they mostly confirmed the things I was already doing”.* |
|  | Gained through alternative sources | P18: *“I figured out how to handle it, but by reading and training. For example, everyone said, “Hold still. Sit still”, but if he doesn't manage it, we can't keep telling him that. Instead, we can say, “Don't hurt your sister”(...). I trained myself, but nobody provided any guidance after the diagnosis”.* |
| Systemic awareness | Developed | P20: “*My daughter's dad lives in a family that's a bit entangled. We are quite hypochondriacal and very anxious, and I believe that living with these dynamics sometimes particularly accentuates an anxious symptomatology in my daughter. She's the only little niece in a family of older people, so she grew up in an environment where she is the center of the world, and this probably contributed to her immaturity from various points of view*”. |
|  | Not developed | P16: “*Irrespectively of the assessment,* *I don't think my son's difficulties depend on family conflicts. I think he would have been the same anyway because even when he was a little boy, he was like that. So, I don't see a correlation*”. |

**Table 4 -** **Effects of the assessment: Emotional aspects**

| **Secondary themes** | **Subthemes** | **Testimonials** |
| --- | --- | --- |
| Proactive | The assessment promoted the grieving process for the loss of an idealized view of the child | P20: *"When you ask for an evaluation for a child, the final assessment itself should be a path toward accepting this disorder and finding serenity because there needs to be acceptance. The entire journey, I believe, should involve guiding the entire family."* |
|  | The assessment promoted the need to repair past errors in parents’ actions | P2: “*I allowed my husband to do many things that maybe I shouldn't have allowed, and that marked my son’s childhood. Not anything dramatic, but phrases that I might have noticed the impact they could have on him. So... I could have done more, but maybe it's a feeling that all parents have, or at least those who question themselves*”. |
|  | Relief | P19: “*In about eighty percent of the cases, the assessment comes when the parent now has awareness that something is wrong. Before this, most of the time, they treat you like you're overreacting, but when the evaluation comes, it gives you a sense of relief. It's like saying, 'Gosh, it's not that I'm wrong, it's not my daughter who's wrong. We weren't wrong when thinking about an assessment to figure out what was happening*”. |
| Hindering | Self-blaming | P18: “*At first, when my son was diagnosed, I thought I might have given him this negative gene*”. |
|  | Shock | P8: “*When you get these things, it's like a cold shower, and you think, ‘Why me? Why him? Why us?’”*. |
|  | Fear for the future | P13: “*The problem is the uncertainty about what happens next because nobody knows what to do with it. It's not like having a problem with a solution, especially in the case of intellectual giftedness. So, what do you do? What do you do with the child's relationships? There is no specific intervention option, such as speech therapy used for learning disabilities, for example*”. |
|  | Sense of shame | P4: “*Not recognizing the pathology in him and not being able to understand it made me feel ashamed, to be honest. I felt ashamed that I didn't get it, that I yelled at him, gave him a smack, and said, “How can you not understand multiplication tables?”*”. |
|  | Sense of inadequacy | P20: “*I think of myself as a bad parent because when I had the first hints, I should have acted immediately. Instead, it took me a year... before getting my daughter evaluated. I feel a little guilty because it took me a while to start*”. |

**Table 5 – Parental perceptions of the relationship with their children’s teachers**

| **Subthemes** | **Testimonials** |
| --- | --- |
| Competence | P3: “*Unfortunately, they are not really prepared, or perhaps not at all, to identify difficulties that go beyond the usual 'he is lazy, he is listless, maybe he has difficulty in some school subject.' In fact, there was something else: there was a learning disorder*”. |
| Empathy and support | P10: *"At kindergarten, these were the only things that the teacher pointed out to me, and I must say that she was good at never scolding him, never imposing anything on him, giving him his own time and space... Then he did what he had to do, and she always rewarded him, saying, 'Well done, well done! Did you see?' She was always able to boost his self-esteem."* |
| Quality of the teacher-child relationship | P19: “*My daughter recalls her school experience as a period of being bullied by her teachers, not by her classmates. She was told things like “You're not good, you're not committed, you're stupid” despite having a memory disorder and a very serious learning disability*”. |
| Adaptation of the teacher’s educational methods to the outcomes of the assessment | P15: *"Unfortunately, the school turns a deaf ear. So, despite my son having specific aids, the school doesn't make much effort. There are teachers who understand his difficulties and vulnerabilities, while others, unfortunately, do not make an effort. Therefore, in listening tests - which my son naturally struggles with in English - the test is like all the others—long, complex, and detailed.”* |
